# Supplementary material for: The Androgen Regulated lncRNA NAALADL2-AS2 Promotes Tumor Cell Survival in Prostate Cancer
Source: Noncoding RNA. 2022 Dec 1;8(6):81. doi: 10.3390/ncrna8060081 (PMC9787508; doi:10.3390/ncrna8060081)
Supplement: Supplementary file 1 [file ncrna-08-00081-s001.zip › ncrna-1968776-supplementary.pdf]

Supplementary Data

Table S1: The average expression of selected lncRNAs in normal prostate, benign prostate, high grade prostate cancer, and CRPC.

lncRNA	Normal prostate	Benign hyperplasia	High grade	CRPC
NAALADL2-AS2	0,00	0,00	0,00	9,92
**RP11-20F24.2**	0,013	0,00	0,50	8,47
**CTD-2126E3.1**	0,14	0,20	0,11	1,64
**CTD-2126E3.3**	0,41	0,21	0,27	3,03
**SNORD57**	108,99	39,82	35,98	168,22
**SNORD3C**	94,61	3,19	4,78	21,06
**RP11-334J6.4**	29,23	1,29	4,49	14,63
**SNHG3**	113,47	25,03	65,36	197,05
**SNORA71A**	30,15	8,71	27,71	74,33
**RNU11**	112,55	20,8	10,74	28,28
**AC012531.25**	0,091	0,03	0,68	1,74
**RN7SL1**	39335,03	58220,24	31405,61	61942,52
**PCGEM1**	0,25	3,37	0,43	0,70
**AC132217.4**	4,94	12,49	2,61	4,22
**PCA3**	0,15	0,05	21,99	2,03

Normal prostate (n=4); benign prostate (n=3); high grade prostate cancer (n=4); CRPC (n=4).

Table S2: Flow cytometry cell cycle histograms.

Experiment | **Non -transfected** | **Reagent control** | **Scramble control** | ***AS2* knockdown**

1

Non -transfected

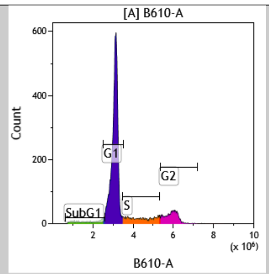

Gate	%Gated
All	100,00
G1	69,94
G2	12,09
S	12,33
SubG1	6,04

Reagent control

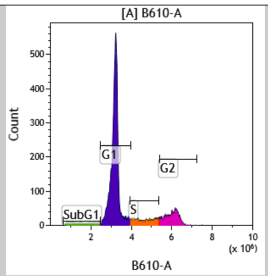

Gate	%Gated
All	100,00
G1	69,41
G2	14,32
S	10,10
SubG1	5,79

Scramble control

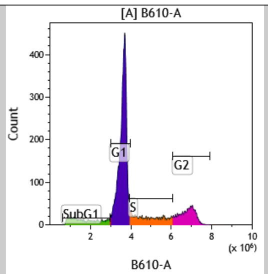

Gate	%Gated
All	100,00
G1	60,65
G2	15,14
S	14,51
SubG1	9,91

AS2 knockdown

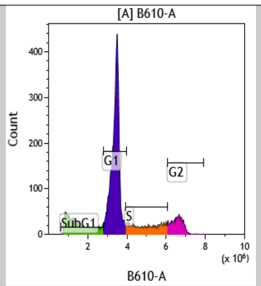

Gate	%Gated
All	100,00
G1	58,23
G2	10,83
S	14,40
SubG1	16,87

2

Non -transfected

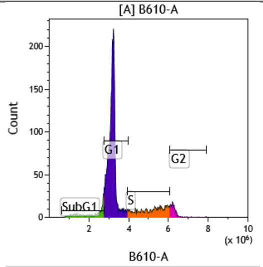

Gate	%Gated
All	100,00
G1	68,29
G2	5,21
S	18,79
SubG1	8,54

Reagent control

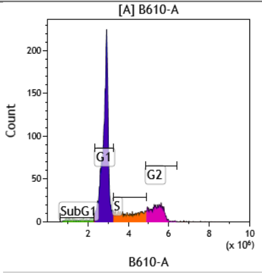

Gate	%Gated
All	100,00
G1	62,02
G2	16,68
S	16,16
SubG1	4,73

Scramble control

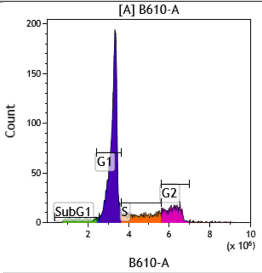

Gate	%Gated
All	100,00
G1	63,92
G2	14,45
S	14,55
SubG1	6,08

AS2 knockdown

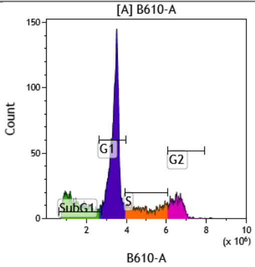

Gate	%Gated
All	100,00
G1	53,66
G2	11,66
S	15,48
SubG1	20,46

3

4

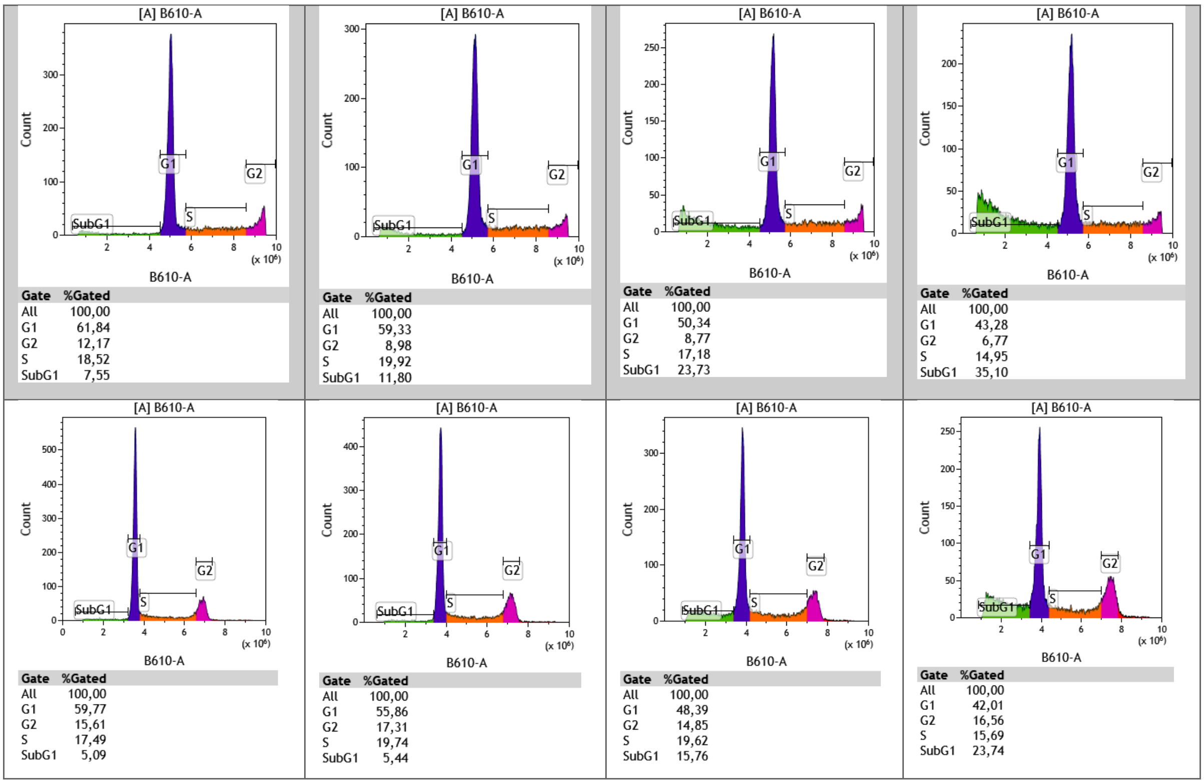

Table S3: Cell culture media.

Cell line	Media	Supplemented nutrients	
LNCaP	RPMI 1640*	2mM L-Glutamine*	Fetal Calf Serum (10%)**
22Rv1	RPMI 1640*	2mM L-Glutamine*	Fetal Calf Serum (10%)**
DuCaP	RPMI 1640*	2mM L-Glutamine*	Fetal Calf Serum (10%)**
LAPC-4	IMDM*	1nM R1881***	Fetal Calf Serum (7.5%)**

Table S4: Primer list.

Gene	Forward primer	Reverse primer
NAALADL2-AS2	5'-ACCAGCGGAAATTGAAAAGCAA-3'	5'-GCTCAGCTCATGTCTTCTCCT-3'
RP11-20F24.2	5'-TTCCAGTGTTTGTCACGTGGTT-3'	5'-TCTAGTTCAGCACCACCCGT-3'
CTD-2126E3.1	5'-TGGCCAACGCCAAGACTTAT-3'	5'-AGACGCGGAGCCTTAAACAG-3'
CTD-2126E3.3	5'-GCCTGACGCCAGAGTTTCT-3'	5'-CAGGCAATTTGCTAGGCTCG-3'
SNORD57	5'-GGAGGTGATGAACTGTCT-3'	5'-GGATCAGGCTCATTAAATC-3'
SNORD3C	5'-CTTGGCATGTCGCGAGAAAG-3'	5'-AATAGGAGGTGCCACACAGC-3
RP11-334J6.4	5'-CCAATAGATCCTTCTGACCCTCC-3'	5'-TCTCTCAGTCACTGTCCTCTTCA-3'
SNHG3	5'-TGCACTTCGCATTTTGGCAT-3'	5'-GCACCTCAATCTTTTGCTCCA-3'
SNORA71A	5'-GCCTGTGCCTAGGTCATTGAT-3'	5'-TAGGGTGGACCCTCCAAACA-3
RNU11	5'-AAGGGCTTCTGTCGTGAGTG-3'	5'-CGGGACCAACGATCACCAG-3'
AC012531.25	5'-CCCTGTGGTGGACAGACTTC-3'	5'-GGGTGGGCTCAACTTTCTCA-3'
RN7SL1	5'-TCCGCACTAAGTTCGGCATC-3'	5'-TCAGCACGGGAGTTTTGACC-3'
PCGEM1	5′-AGATGCACTGGGACTCAACG-3′	5′-CCCTAGGAGTAGGCCTGTGT-3
AC132217.4	5'-CTGCCCATCGACCAGGTTTG-3'	5'-CACGGGGAGGAGACAAGATG-3'
PCA3	5'-AGGGGAGATTTGTGTGGCTG-3'	5'-ATGTCCTTCCCTCACAAGCG-3'
AR	5'-AAGGAACTCGATCGTATCATTGC-3'	5'-TTGGGCACTTGCACAGAGAT-3'
NAALADL2	5'-CCTAACACGTCTTTGCAAGGTAA-3'	5'-CTAAGTCAAGGGCAGTGGCT-3'
KLK3	5'-CGGTTGTCTTCCTCACCCTG-3'	5'-CCTCCCACAATCCGAGACAG-3'
GATA2	5'-AAGAGTCCGCTGCTGTAGTC-3'	5'-AGGCCTAGCTACTATGGGCA-3'
FOXA1	5'-AGGGCATGAAACCAGCGAC-3'	5'-GAGTTCATGTTGCTGACCGGG-3'
GAPDH	5'-TCAAGGCTGAGAACGGGAAG-3'	5'-TGGACTCCACGACGTACTCA-3'

Table S5: Probe set used to image *NAALADL2-AS2*.

Probe #	Probe (5'-> 3')	Probe position	Percent GC
1	gagattgctttgatgtctca	26	40.0%
2	attcagtctttccagaacta	48	35.0%
3	ctcatttcagcagatgctgc	70	50.0%
4	ccgctggtgaacattgatta	93	45.0%
5	ccatctctttgcttttcaat	115	35.0%
6	cttgagtggatccttggaat	140	45.0%
7	agctcatgtcttctcctaaa	205	40.0%
8	tttcaacctcttctttatcc	235	35.0%
9	tactctgtctaagccatacg	258	45.0%
10	aagactctaaaagttacccc	299	40.0%
11	tcagaagctttggccttaag	321	45.0%
12	gaaggcaactgtcactcttt	348	45.0%
13	tattatgctctttctggcag	408	40.0%
14	atttctactgtaggactcat	446	35.0%
15	caccttgaaatgtcttacca	503	40.0%
16	ttttagccaaagagctgcat	534	40.0%
17	cagcatcgtttctattctgt	571	40.0%
18	tgctcgttcctaatagatct	629	40.0%
19	cctttctatggactagtttt	878	35.0%
20	acgtgtaattggtcaacagt	900	40.0%

Designed by Biosearchtech.

Table S6: GapmeR and siRNA oligos.

Name	siRNA/LNA	Sequence	Supplier
SCR (Control)	LNA	[C*C*U*U*C*]C*C*T*G*A*A*G*G*T*T*[C*C*U*C*C]	Eurogentec
AS2_IDT	LNA	A*U*A*-A*C*5*-7*8*5*-5*5*8*-7*6*8*-C*G*U*-U*C	Eurogentec
siFOXA1	siRNA	ACAUGACCAUGAACACCAUtt	Ambion, Silencer Select
siGATA2	siRNA	GGCGCACAACUACAUGGAAtt	Ambion, Silencer Select
SCR (Control)	siRNA	Ambion Silencer Select® Negative Control #2 (P/N AM4613)	Ambion, Silencer Select

Figure S1: Somatic alterations of *NAALADL2* and *TBL1XR1* in prostate cancer.

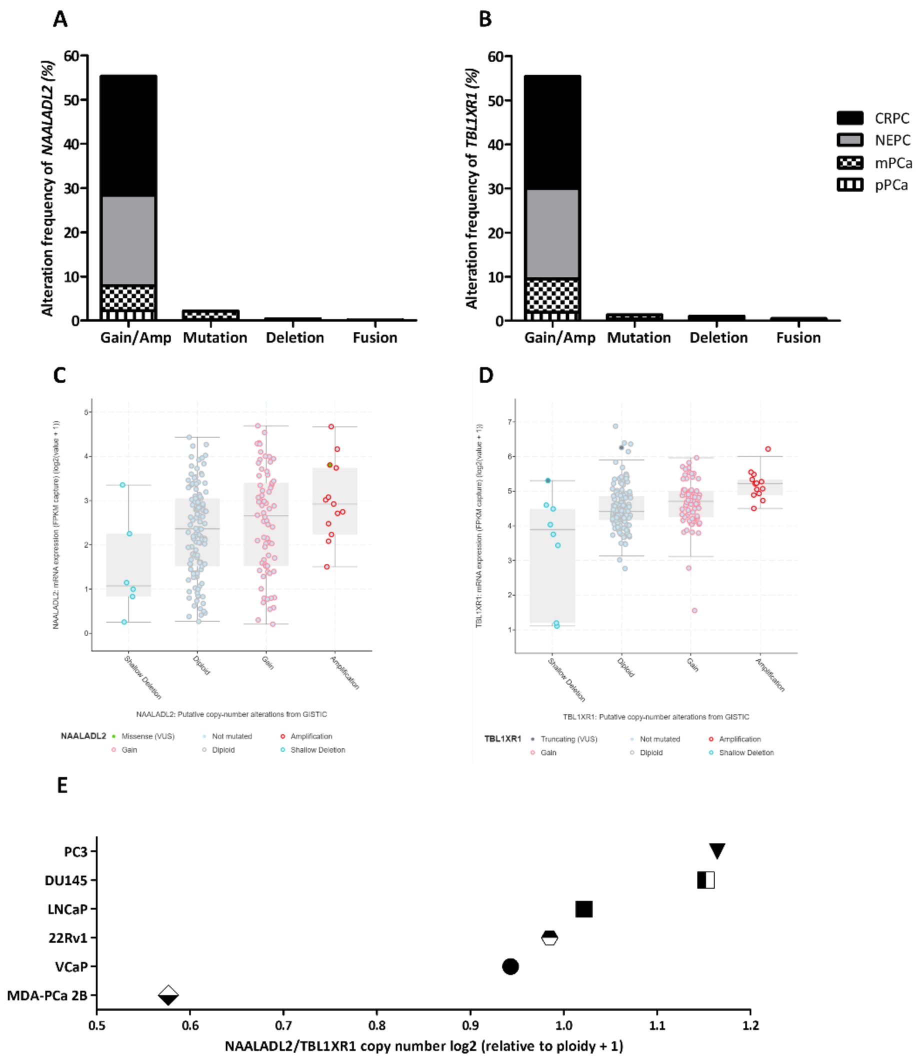

Alteration frequency of *NAALADL2* (A) and *TBL1XR1* (B) in prostate cancer subtypes. Copy number alterations and relative mRNA expression levels of *NAALADL2* (C) and *TBL1XR1* (D) in PCa. Copy numbers of *NAALADL2* and *TBL1XR1* in 6 common prostate cancer cell lines (E). Publicly available data containing 3804 patients in 16 non-overlapping studies consisting of 11 primary PCa (pPCa), 4 metastatic PCa (mPCa), 1 neuroendocrine PCa (NEPC) and castration resistant PCa (CRPC) (A/B) [1]. The copy number and mRNA plots were produced using cBioportal including 2 studies (C/D) [2,3]. Copy number alterations in cell lines were obtained from the Broad Institute's depmap portal (https://depmap.org/portal/ccle/).

Figure S2: Relative expression of *NAALADL2-AS2*, *NAALADL2*, *KLK3*, and *AR* in LAPC-4 and LNCaP.

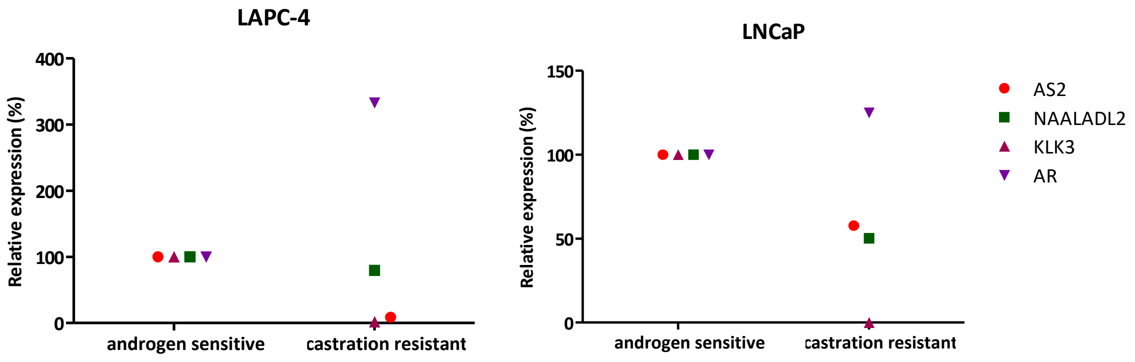

Data was downloaded from the Gene Expression Omnibus (GSE178963).

Figure S3: *FOXA1* has a transcriptional start site at the *NAALADL2-AS2* locus.

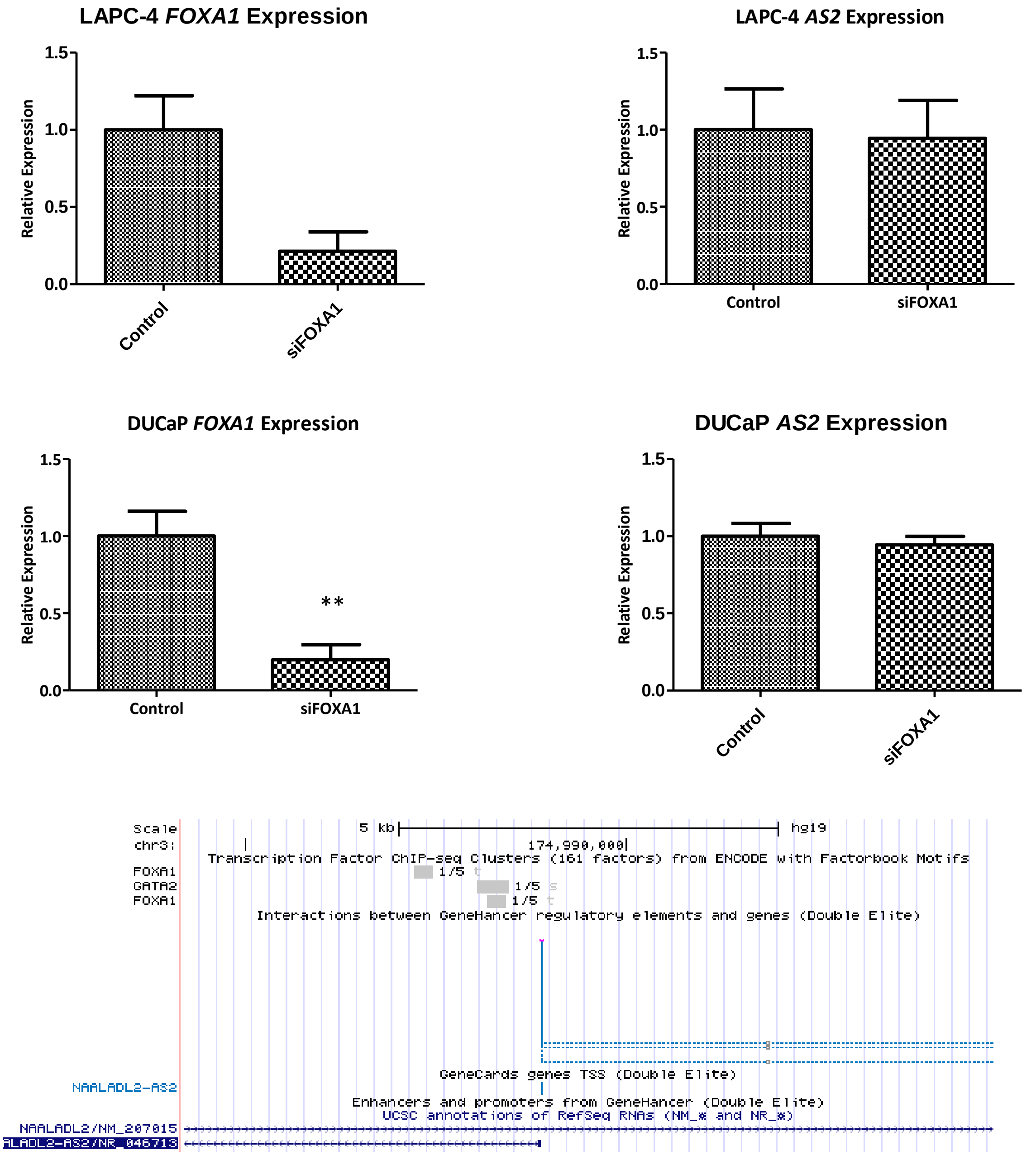

Genomebrowser was used to generate the bottom figure (http://genome.ucsc.edu).

Figure S4: Quantitative PCR validation of *NAALADL2-AS2* regulated genes.

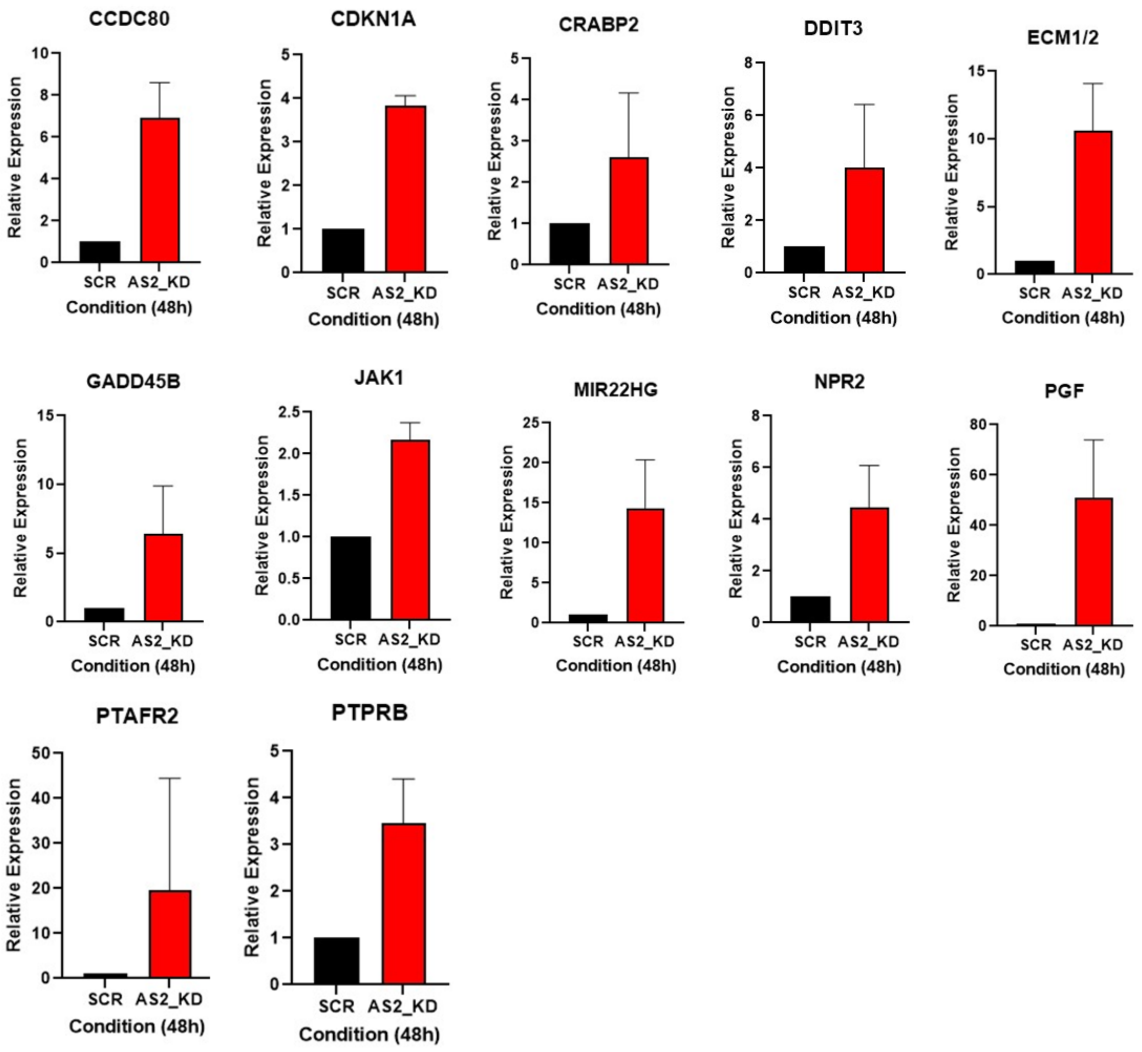

All data shown is from triplicate qPCR assays.

References

1. Simpson, B.S.; Camacho, N.; Luxton, H.J.; Pye, H.; Finn, R.; Heavey, S.; Pitt, J.; Moore, C.M.; Whitaker, H.C. Genetic Alterations in the 3q26.31-32 Locus Confer an Aggressive Prostate Cancer Phenotype. *Commun Biol* **2020**, *3*, doi:10.1038/s42003-020-01175-x.

2. Beltran, H.; Prandi, D.; Mosquera, J.M.; Benelli, M.; Puca, L.; Cyrta, J.; Marotz, C.; Giannopoulou, E.; Chakravarthi, B.V.S.K.; Varambally, S.; et al. Divergent Clonal Evolution of Castration-Resistant Neuroendocrine Prostate Cancer. *Nat Med* **2016**, *22*, 298–305, doi:10.1038/nm.4045.

3. Weinstein, J.N.; Collisson, E.A.; Mills, G.B.; Shaw, K.R.M.; Ozenberger, B.A.; Ellrott, K.; Sander, C.; Stuart, J.M.; Chang, K.; Creighton, C.J.; et al. The Cancer Genome Atlas Pan-Cancer Analysis Project. *Nat Genet* **2013**, *45*, 1113–1120, doi:10.1038/ng.2764.
